# Potential Effects of Nichi Glucan as a Food Supplement for Diabetes Mellitus and Hyperlipidemia: Preliminary Findings from the Study on Three Patients from India

**DOI:** 10.1155/2012/895370

**Published:** 2012-12-10

**Authors:** Vidyasagar Devaprasad Dedeepiya, Gurusamy Sivaraman, Athi P. Venkatesh, Senthilkumar Preethy, Samuel J. K. Abraham

**Affiliations:** ^1^Division of Translational Medicine, Nichi-In Centre for Regenerative Medicine (NCRM), PB 2278, Tamil Nadu, Chennai 600094, India; ^2^Arogya Siddha Hospital, A-2 Alankar Plaza, 425 Kilpauk Garden Main Road, Chennai 600 010, India; ^3^Kingwood Psychiatry, 19701 Kingwood Dr., Building No. 3, Kingwood, TX 77339, USA; ^4^Hope Foundation (Trust), B6, 13, Zakariah colony III St., Choolaimedu, Chennai 600094, India; ^5^Department of Clinical Research, School of Medicine, University of Yamanashi, 1110, Shimokato, Yamanashi, Chuo 409-3898, Japan

## Abstract

Beta Glucan food supplements have been reported to be of benefit in diabetes and hyperlipidemia. We report a pilot study of the effects of Nichi Glucan, 1, 3-1, 6 Beta Glucan food supplement, in lowering the blood glucose and lipid levels in three patients with noninsulin-dependent diabetes mellitus (NIDDM) from India. These patients had increased blood glucose and lipid levels inspite of routine antidiabetic and lipid level lowering medications. Each of the participants took 1.5 g of Nichi Glucan per day with food for two months along with their routine medications. The relevant parameters to assess glycemic status and lipid levels were calculated at the baseline and at the end of two months. After two months of continuous consumption, in one patient, the HbA1c decreased from 9.1% to 7.8%, and the glycemic target of HbA1c <6.5% laid down by the International Diabetes Federation was reached in two patients. Lipid levels also decreased significantly. Based on our findings, Nichi Glucan food supplement can be considered along with routine medications in patients with Type II diabetes with hyperlipidemia. Further studies are needed to validate the results.

## 1. Introduction

The rampantly increasing incidence of lifestyle changes has contributed to the massive increase in the prevalence of diabetes with nearly 51 million people suffering from Diabetes in India [1]. There is also growing concern on the term “Asian Indian Phenotype,” which refers to certain unique clinical and biochemical abnormalities in Indians, including an increased insulin resistance, greater abdominal adiposity, dyslipidemia with low HDL cholesterol, elevated serum triglycerides and increased small, dense LDL cholesterol, which further with an increased ethnic susceptibility makes Indians at high risk for diabetes and premature coronary artery disease [1]. Studies indicate that 31.4% of the population in South India have abdominal obesity, 45.6% have hypertriglyceridemia, 65.5% have low HDL, 55.4% have hypertension, and 26.7% have raised fasting plasma glucose, all of which are major features of the metabolic syndrome (MetS) [2]. Type II Diabetes or the noninsulin-dependent diabetes mellitus (NIDDM) is the more common type of diabetes with its prevalence particularly higher in South India compared to other parts of India [3]. In addition to the influence on health, the toll of diabetes on the country's economy is alarming with nearly 2.1% of the nation's GDP spent on treatment for diabetes [4]. In this regard, apart from therapeutic interventions such as oral glucose lowering drugs and insulin, dietary supplements are a potential intervention, both preventive and therapeutic. In this context, Beta Glucan-based food supplements have been developed for the treatment of various diseases including diabetes, hyperlipidemia, cancer, and infectious diseases with promising results, based on clinical studies [5]. Beta Glucans are polysaccharides with glucose residues joined by beta linkage found in the cell wall of certain fungi, yeast, oat, barley, bacteria, and so forth [5]. However, such Beta Glucan-based food supplements for lowering glucose levels and treating metabolic syndrome have not gathered prominence in India. Herein, we report the effects of a food supplement, Nichi Glucan, which is 1, 3-1, 6 Beta Glucan, in lowering the blood glucose and lipid levels on the basis of results obtained from a preliminary study involving three patients with NIDDM from South India.

## 2. Materials and Methods

### 2.1. The Nichi Glucan

The Beta Glucan used in the study is Nichi Glucan, a commercially available, water soluble 1, 3-1, 6 Beta Glucan obtained from the cultured black yeast (*Aureobasidium pullulans*), which is an approved health food supplement in Japan. The Nichi Glucan contains 5%  *β*-1, 3-1, 6-glucan, 71% starch, 13% corn starch, 13% Aureobasidium nutrient solution as thickening agent, 2% tricalcium phosphate, and 1% pullulans as manufacturing solutions. The aqueous solutions are used during the manufacturing process; however, the above percentages were calculated based on the dry weight of the product. The Nichi Glucan was supplied by GN Corporation through Nichi-Vision Life Sciences (NVLS), India. It was given to the participants of the study in the form of sachets each containing 0.5 g granules of Nichi Glucan. The participants were advised to take three of such sachets per day, one at morning, one at afternoon, and one at night as a food supplement for two months. The HbA1c, fasting and postprandial blood glucose levels, serum cholesterol, LDL, HDL, and triglyceride levels were calculated before starting the Nichi Glucan supplement and at the end of two months. 

### 2.2. Description of Participants Included in the Study

Three participants were included in the study. All three were from South India. One of them (Patient III) was a vegetarian on all days but could consume all kinds of milk products and the other two (Patients I and II) were vegetarian on selected days but could consume milk products on all days and nonvegetarian food such as egg products, chicken, fish, and lamb as animal proteins on other days. Food intake and physical activity were quantified at the baseline by a physician. All three participants had undergone individual counselling by a common physician during the study period concerning daily food intake, and they were advised to consume a diet similar to their consumption prior to the starting of the Nichi Glucan intake. All three participants did only mild exercise such as walking for 30 minutes, simple non-strenuous domestic work, and travelling by public transport. The participants were advised not to change their diet habits or physical activity during the study. Every two weeks during the study, the food intake and physical activity were evaluated by the same physician who evaluated them before the start of the study.

#### 2.2.1. Patient I

A 46-year-old male, white collar job holder, BMI of 24.2, with history of hypertension for 10 years, and has Type II diabetes for the past 10 years. He has been taking insulin injection, 12 units at morning and night for past six years. He has also been prescribed Metformin 500 mg one tablet at morning and night and 1000 mg at noon and Pioglitazone 15 mg twice daily. He is not a smoker but consumes alcohol occasionally. He also had an elevated lipid profile, for which he was prescribed Atorvastatin 40 mg and Fenofibrate 67 mg twice daily. He started taking Nichi-Glucan as a food supplement in the dosage described above, on the 20th November 2011, and his parameters of relevance were measured before and after taking Nichi-Glucan for two months. During this period of two months, he did not change his food habits or other lifestyle related events. His antiglycemic medications were also continued as before.

#### 2.2.2. Patient II

A 60-year-old female, house wife, BMI of 26.6, with history of hypertension for five years, under medication with Amlodipine 5 mg once daily, and Type II diabetes for 1 year. She has been taking oral antidiabetic agent (Metformin) 500 mg thrice daily after food for the past one year. She had an elevated lipid profile, for which she was prescribed lipid-lowering drugs, but she did not follow it due to side effects of nausea and vomiting after taking the drugs initially. She started taking Nichi Glucan as a food supplement in the dosage described above, on 28th November 2011, and her parameters of relevance were measured before and after taking Nichi Glucan for two months. During this period of two months, she did not change her food habits or other lifestyle related events. Her antidiabetic medications were also continued as before.

#### 2.2.3. Patient III

A 63-year-old female, house wife, with a BMI of 26.1, with Type II diabetes for the past one year and on insulin 16 units in the morning and 12 units in the night along with Metformin 500 mg thrice daily and Pioglitazone 15 mg twice daily, started taking Nichi Glucan as a food supplement on 26th November 2011, and her parameters of relevance were measured before and after taking Nichi Glucan for two months. She is neither a smoker nor consumes alcohol. During this period of two months, she did not change her food habits or other lifestyle related events. Her antidiabetic medications were also continued as before.

The study was done in accordance with the Helsinki Declaration, and all the participants were included in the study after proper informed consent.

## 3. Results

The HbA1c levels and fasting, postprandial blood glucose levels before and after taking Nichi Glucan are depicted in Table 1. It can be observed that in spite of the antidiabetic medication, the glycemic target, HbA1c <6.5%, laid down by the International Diabetes Federation [5] was not achieved in any of the patients prior to Nichi Glucan supplementation. However, after Nichi Glucan supplementation, there is a decrease in the blood glucose levels in all of the three patients with the glycemic target reached in two of the patients (Patient II—HbA1c—5.9% and Patient III—HbA1c—5.6%). A decrease in total cholesterol, LDL, and triglycerides and an increase in HDL levels can also be appreciated after Nichi Glucan supplementation.

## 4. Discussion

Diabetes has a global prevalence of 366 million in 2011, and this figure is expected to increase to 552 million by 2030 [6]. Current treatments of Diabetes both Types I and II include diet modifications, exercise, insulin injection, and glucose-lowering drugs [7]. Metabolic syndrome, which includes diabetes, obesity, and hyperlipidemia [8], is another facet of consideration, particularly in Indian diabetic patients. With the increasing evidence of the Asian Indian Phenotype in whom, diabetes sets in at least a decade earlier compared to their western counterparts [9], the need to control the glycemic level and hyperlipidemia in Indians becomes increasingly more important. At this juncture, the role of food supplements in reducing the blood glucose and lipid levels needs attention.

There are several literatures which have reported the efficacy of Beta Glucan food supplements in lowering blood glucose levels, improving glucose tolerance, decreasing hyperlipidemia, improving immunity, and decreasing infections [10–13]. Antitumour properties of Beta Glucans have also been reported [14]. Beta Glucans are obtained from a variety of sources like fungi, mushrooms, oats barley, and other bacteria. Fungal Beta Glucans have 1, 3-1, 6 linkages while those from oats and barley have 1, 3-1, 4 linkages [5]. The Beta Glucan used in this study was obtained from a black yeast known as *Aureobasidium pullulans* (Strain AFO-202) isolated by Dr. Ikewaki et al., which secretes the 1, 3-1, 6 Beta Glucans extracellularly in the culture medium. Hence refining and purification are relatively simple [12]. Further, it is odorless, water soluble, and does not change the taste or flavor of the food thus making it an ideal food supplement. It can also be used as a food additive due to its water retentive and thickening characteristics. This Beta Glucan is already available as a commercial health food supplement, but the efficacy of the same in Type II diabetes patients in India has not been yet reported.

**Figure 1:**
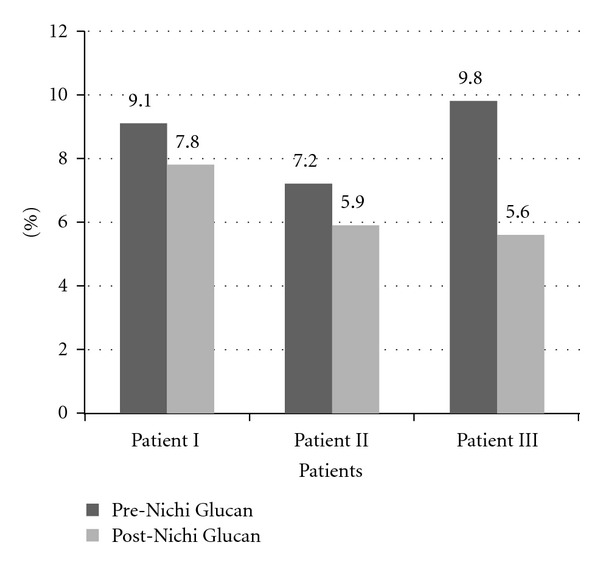
HbA1c Levels before and after food supplementation with Nichi Glucan.

From the results of this study, it can be observed that there is a significant decrease in the glycemic levels in all of the three patients, wherein in the first two patients, the HbA1c levels decreased by 1.3%, while in the third patient, the HbA1c level decreased by a significant 4.2% (Figure 1). The fasting and postprandial blood glucose levels also decreased considerably. A consensus article by the American Diabetes Association and the European Association for the Study of Diabetes states that the expected decrease of HbA1c levels with Metformin and Thiazolidinedione monotherapy is 1%-2%, and with Insulin a decrease of 1.5%–3.5% can be expected [5]. In all these three patients, before supplementation with Beta Glucan, the glycemic target of HbA1c <6.5% was not achieved, and plasma glucose levels were high in spite of intake of antidiabetic medication including insulin injections in two of the patients, but after supplementation glycemic target was achieved in two of the patients. Plasma Glucose levels also returned to the normal range. The average decrease in the levels before and after Beta Glucan supplementation of the total cholesterol (2.05 mmol/L), LDL (1.91 mmol/L) and Triglycerides levels (1.04 mmol/L) is comparable or even slightly higher than the levels reported in other literatures including a meta-analysis on changes in the lipid profile after Beta Glucan consumption [15, 16]. The HDL cholesterol levels also increased after Nichi Glucan supplementation which is another favorable response. As the patients did not change their food habits or physical activity during the study, the influence of these factors on the reduction of HbA1c levels and total cholesterol, LDL, and Triglyceride levels may be considered negligible or minimal.

The total quantity of Nichi Glucan consumed was only 1.5 g per day, and since the three subjects involved in the study were used to a relatively fibre dominant south Indian diet, this 1.5 g in addition to the diet did not amount to a significant proportion leading to any high fibre-related side effect. Also in a study done in 27 elderly people aged above 70 years, it has been confirmed that an oral administration of nearly 150 mg/day of *β*-1, 3-1, 6-glucan similar to the one used in the study, through gastronomy or nasal tube, for a period of 3 months, did not have any adverse changes on the levels of Na, K, Cl, glucose, uric acid, total cholesterol, LDL, HDL, triglyceride, total protein, albumin, and cholinesterase, *γ*-GTP, AST, ALT, and CRP in the blood of the subjects as revealed by biochemical screening [17].

Our study has limitations of any observational study based on few patients. Hence, our findings need to be interpreted with caution. However, on the basis of limited information available from three patients, we hypothesize that three-time intakes of 0.5 g Nichi Glucan daily can serve as an effective food supplementation to diabetics with particular relevance to Indians who have concomitant risk of development of metabolic syndrome and cardiovascular disease associated with diabetes. However, this needs evidence from large randomized controlled trials in such patients.

## 5. Conclusion

Daily food supplementation with 1, 3-1, 6 Beta Glucan (Nichi Glucan) along with routine antidiabetic and lipid-lowering drugs has favorable response in improving glycemic status and lipid status in Indian Patients with Type II Diabetes with hyperlipidemia. Further studies with larger samples are needed to validate the results.

## Figures and Tables

**Table 1:** Characteristics of patients and study parameters of relevance before and after food supplementation with Nichi Glucan.

Characteristics	Patient I		Patient II		Patient III	
Age	46		60		63	
Sex	M		F		F	
BMI	24.2		26.6		26.1	
Number of years with Type II diabetes	10		1		1	

Parameters	Baseline	At the end of the study	Baseline	At the end of the study	Baseline	At the end of the study

HbA1c (%)	9.1	7.8	7.2	5.9	9.8	5.6
Fasting blood glucose level (mg/dL)	171	106	114	103	129	85
Post prandial blood glucose level (mg/dL)	244	203	192	111	252	118
Total serum cholesterol (mmol/L)	7.3	4.31	7.65	5.09	4.008	3.38
LDL (mmol/L)	4.6	2.48	5.81	3.33	2.56	1.42
HDL (mmol/L)	1.008	1.29	0.98	1.11	0.9	1.29
Triglycerides (mmol/L)	3.29	1.17	1.84	1.38	1.18	0.62
